# Experimental Study on the Behavior of Galvanized Steel Elliptical Tubes with Different Major-to-Minor Axis Length Ratios Under Cyclic Bending with Various Curvature Ratios

**DOI:** 10.3390/ma19051043

**Published:** 2026-03-09

**Authors:** Chia-Ling Sung, Wen-Fung Pan

**Affiliations:** Department of Engineering Science, National Cheng Kung University, Tainan 701, Taiwan; n98131075@gs.ncku.edu.tw

**Keywords:** galvanized steel elliptical tubes, major-to-minor axis length ratios, cyclic bending, curvature ratios, moment, curvature, minor-axis variation, number of cycles to buckling

## Abstract

Although the cyclic bending behavior of circular and elliptical steel tubes has been widely studied, the combined effects of major-to-minor axis length ratio and curvature ratio on the deformation characteristics and buckling life of galvanized steel elliptical tubes remain insufficiently understood. This study experimentally investigates the cyclic bending response and failure behavior of galvanized steel elliptical tubes with major-to-minor axis length ratios of 1.5, 2.0, 2.5, and 3.0 under curvature ratios of −1, −0.5, and 0. The curvature ratio is defined as the minimum controlled curvature divided by the maximum controlled curvature. Buckling is defined as the cycle at which a pronounced 20% drop in peak bending moment is observed. The response is characterized by moment (N⋅m)–curvature (m^−1^) hysteresis and minor-axis variation with curvature, while failure is evaluated using the relationship between curvature range and number of cycles to buckling. The results show that stable elastoplastic hysteresis loops develop for all curvature ratios, with slight cyclic relaxation observed at curvature ratios of −0.5 and 0. Increasing the axis length ratio slightly reduces the peak moment under a fixed curvature ratio. Minor-axis variation increases progressively with cycle number, exhibiting serrated curves at an axis ratio of 1.5 and butterfly-shaped curves at higher axis ratios. Symmetric behavior is observed at a curvature ratio of −1, whereas asymmetric responses occur at −0.5 and 0. The failure results indicate that larger curvature ranges and higher axis length ratios reduce the number of cycles to buckling, while curvature ratios closer to −1 enhance buckling life. On a log–log scale, the relationship between curvature range (m^−1^) and number of cycles to buckling becomes linear. A theoretical model is proposed and shows good agreement with the experimental results.

## 1. Introduction

Traditional circular tubes have been extensively employed as pipelines and structural support members because of their geometric simplicity, manufacturing convenience, and widespread industrial standardization [1,2]. Despite these advantages, circular tubes often exhibit relatively limited load-carrying capacity, wear resistance, and energy dissipation capability when subjected to complex loading conditions, particularly under bending and cyclic loads [2,3]. Such loading scenarios tend to induce cross-sectional ovalization, local buckling, and progressive stiffness degradation [4,5,6], which significantly compromise structural performance and durability.

A substantial body of experimental and analytical research has been devoted to understanding the monotonic and cyclic bending response of circular tubes, reflecting their widespread structural applications and their susceptibility to bending-induced instability. Among these efforts, Kyriakides and Shaw [2] developed a dedicated mechanical testing apparatus capable of imposing well-controlled monotonic and cyclic bending on circular tubes, both in the presence and absence of external pressure. This apparatus enabled precise control of curvature history and loading conditions, thereby providing a reliable experimental framework for systematically investigating bending-induced deformation and instability phenomena.

Owing to its robustness and versatility, this experimental setup has been extensively adopted in subsequent studies to examine the bending behavior of circular tubes fabricated from a broad range of materials, including 1020 and 1018 carbon steels, 304 stainless steel, 6061-T6 aluminum alloy, and NiTi alloys (Kyriakides and Shaw [2]; Corona and Kyriakides [4,7]; Corona et al. [8]; Limam et al. [9,10]; Bechle and Kyriakides [11]; Jiang et al. [12]; Kazinakis et al. [13]). These investigations have consistently demonstrated that bending-induced ovalization plays a critical role in governing stiffness reduction, the onset of local buckling, and progressive degradation under cyclic loading. As a result, they have provided fundamental insights into the coupled deformation mechanisms and failure processes of circular tubes subjected to repeated bending.

Beyond metallic hollow tubes, considerable research efforts have also been directed toward the bending and buckling behavior of composite and filled tubular systems, motivated by the need to enhance structural efficiency and stability through material and sectional hybridization. Yuan and Mirmiran [14] studied fiber-reinforced concrete-filled plastic pipes, showing that composite interaction enhances load transfer and buckling resistance. Elchalakani et al. [15] conducted cyclic bending tests on cold-formed circular hollow sections, establishing slenderness limits for fully ductile behavior under repeated loading. Houliara and Karamanos [16] examined circular tubes under combined bending and external pressure, highlighting the coupling effects on instability. Elchalakani and Zhao [17] evaluated cyclic bending of concrete-filled steel tubes, demonstrating improved stiffness retention and energy dissipation due to composite action. Zhi et al. [18] analyzed single-layer cylindrical shells under seismic excitation, revealing that cyclic and transient loading affect bending instability onset and evolution. Yazdani and Nayebi [19] investigated pipes under periodic bending with internal pressure, showing that pressure–bending interaction accelerates damage. Guo et al. [20] studied thin-walled circular hollow tubes, highlighting the influence of wall slenderness on deformation and stability. Shariati et al. [21] examined SS316L cylindrical shells under cyclic bending, providing evidence of material-specific cyclic degradation.

Elchalakani et al. [22] proposed ductile slenderness limits for concrete-filled tubes based on bending tests. Shamass et al. [23] studied circular tubes under non-proportional loading, showing that load sequence affects instability onset. Li and Wang [24] investigated seismic instability in cable-reinforced reticulated shells, emphasizing stability under multi-directional dynamic loading. Chegeni et al. [25] examined curved pipes under internal pressure, demonstrating sensitivity of bending resistance to corrosion and geometric degradation. Jin et al. [26] analyzed local buckling of circular tubes under combined axial and bending loads, clarifying the role of cross-sectional slenderness. Silveira et al. [27] applied the constructal law to square steel plates with elliptical perforations, providing guidance for shape optimization under biaxial compression. He et al. [28] proposed a corrected relation between bending angle and transition-section length in AA5052 aluminum tubes, validated by experiments and simulations.

Wang et al. [29] studied concrete-filled square steel tubes with internal angles under four-point bending, identifying key parameters affecting flexural performance. Liu et al. [30] investigated multicell concrete-filled round-ended steel tubes, showing ductile failure and strong composite action. Yang et al. [31] developed a damping strain energy model for CFRP hollow tubes, highlighting laminate effects on bending and torsional damping. Wang et al. [32] proposed a framework for bending–torsion buckling in spatial tube forming, emphasizing combined loading, geometry, and material imperfections. Park et al. [33] analyzed bending effects on helical SMR tubes, showing that tube thickness influences ovality, eccentricity, and collapse pressure. Lin and Pan [34] carried out experimental investigations on the progressive deformation behavior and the associated critical change in the outer minor-axis length of 6063-T5 aluminum alloy elliptical-square tubes under bending.

Although the cyclic bending behavior of circular and elliptical steel tubes has been investigated in previous studies, the existing literature has primarily focused on single geometric parameters or specific loading conditions. In particular, for elliptical tubes, the combined influence of major-to-minor axis ratio and curvature ratio on cyclic deformation evolution and buckling life has not been systematically examined. Due to the inherent geometric asymmetry of elliptical sections, the stiffness distribution and ovalization behavior differ fundamentally from those of circular tubes, leading to distinct cyclic instability mechanisms. However, the interaction between minor-axis deformation, curvature amplitude, and fatigue-type buckling response remains insufficiently quantified.

In practical use, elliptical tubes are frequently subjected to cyclic bending loads, under which their bending rigidity gradually degrades as the applied load or number of cycles increases. The evolution of the short-axis dimension has been shown to serve as a reliable indicator of this degradation, while buckling or local instability may occur once a critical threshold is exceeded. Compared with circular tubes, the non-uniform curvature and anisotropic geometry of elliptical cross-sections introduce more complex deformation mechanisms, including coupled ovalization, localized stiffening, and asymmetric buckling patterns. Despite the growing adoption of elliptical tubes in engineering practice, systematic experimental and analytical investigations into their bending behavior, cyclic degradation mechanisms, and instability characteristics remain limited, leaving an important gap in understanding their mechanical performance under realistic service conditions.

In 2023, Yu and Pan [35] conducted experimental investigations on SUS304 stainless steel elliptical tubes with four different major-to-minor axis length ratios, subjecting them to cyclic bending at four orientation angles until buckling occurred. Building on this prior work, the objective of the present study is to systematically investigate the combined effects of major-to-minor axis length ratio and curvature ratio on the cyclic bending response and buckling behavior of galvanized steel elliptical tubes. Particular emphasis is placed on (i) the evolution of minor-axis deformation, (ii) the relationship between curvature range and number of cycles to buckling, and (iii) the development and validation of a predictive theoretical model. The results are expected to provide a clearer mechanistic understanding of cyclic degradation and instability in elliptical tubes and to offer practical guidance for structural design and reliability assessment.

## 2. Experiment

Cyclic bending tests were conducted on elliptical tubes with varying major-to-minor length axis length ratios under different curvature ratios, using a tube-bending machine coupled with a curvature–ovalization measurement apparatus. This setup enabled precise control of bending conditions while simultaneously capturing the evolution of cross-sectional deformation. The materials, specimen configurations, and detailed testing procedures are described in the following sections, providing a comprehensive foundation for the systematic investigation of bending response, minor-axis variation, and instability mechanisms in elliptical tubes under repeated loading.

### 2.1. Bending Device

**Figure 1:**
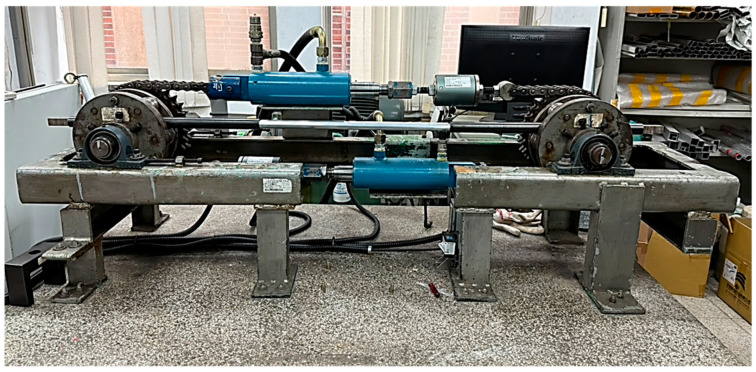
Photograph showing the tube-bending machine used in the experiments [34].

Cyclic bending tests on galvanized steel elliptical tubes were conducted using a four-point bending machine, as shown in Figure 1. The equipment consists of two rotating sprockets mounted on a pair of supporting beams, around which a heavy-duty chain is looped and guided by robust structural supports. This design accommodates specimens up to 1 m in length, while allowing free axial movement of the tube as it interacts with the rollers during bending.

Bending loads are applied through two concentrated forces transmitted via a pair of rollers. Pure bending is achieved by retracting either the upper or lower cylinder, causing the sprockets to rotate and induce a uniform curvature in the specimen. Reverse bending is accomplished by reversing the flow direction of the hydraulic circuit, enabling cyclic loading under well-controlled curvature conditions.

This configuration provides precise control over both forward and reverse bending cycles, allowing accurate measurement of the moment–curvature response, minor-axis variation, and progressive structural degradation of the tubes. The setup ensures reproducibility and reliability, making it suitable for systematic investigation of the effects of cross-sectional geometry and curvature ratios on the cyclic bending behavior and instability of galvanized steel elliptical tubes.

### 2.2. Curvature–Ovalization Measurement Apparatus (COMA)

**Figure 2:**
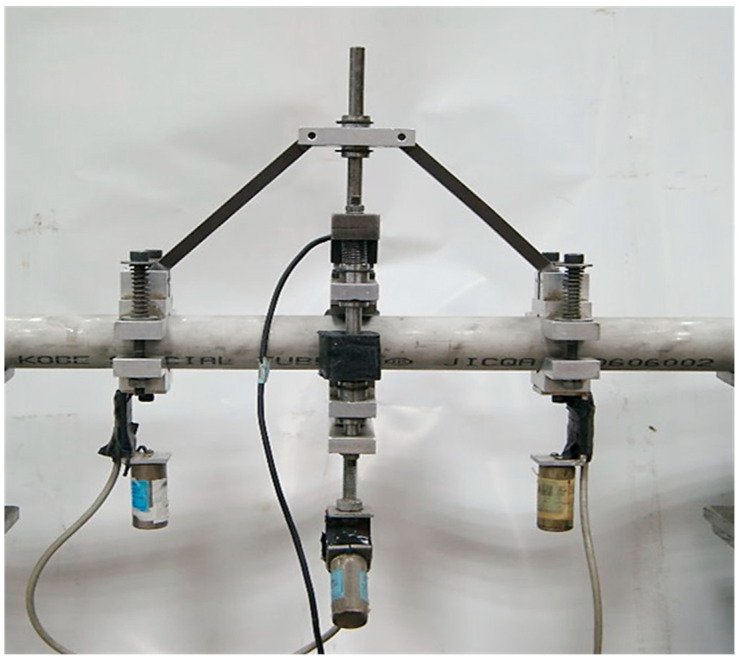
Photograph showing the COMA used in the experiments [34].

Figure 2 presents the curvature–ovalization measurement apparatus (COMA) developed by Pan et al. [36], employed to measure tube curvature and cross-sectional ovalization. Ovalization is defined as the relative change in outer diameter, calculated as the difference between the deformed and original diameters divided by the original diameter. The lightweight device was positioned near the tube’s mid-span, with curvature determined from the fixed spacing between two side-mounted inclinometers and the angular displacements recorded by these sensors. A magnetic detector at the COMA center enabled precise measurement of diameter changes in circular tubes. For the present study, the COMA was adapted to monitor minor-axis variations in elliptical tubes, providing high-resolution data for assessing cross-sectional deformation and the progression of bending-induced degradation.

### 2.3. Elliptical Tubes

The experiments employed hot-dip galvanized steel elliptical tubes, with Q235 carbon structural steel as the base material and a Z120 grade zinc coating of approximately 17 μm thickness. All tubes used in this study were welded. The chemical compositions of the base steel and the galvanized coating were measured using a SpectroMaxx LMF optical emission spectrometer (Spectro Analytical Instruments, Kleve, Germany). The steel tubes were hot-dip galvanized following standard procedures: the specimens were first cleaned and pickled in dilute acid to remove surface oxides, and then dipped in molten zinc at 450 °C for 3 min, resulting in the Z120 grade coating. After galvanization, the tubes were cooled in ambient air. The chemical compositions are summarized in Table 1 and Table 2, while Table 3 lists the mechanical properties of the galvanized tubes. All square tubes had a fixed length of 500 mm and a uniform wall thickness of 1 mm. The mechanical properties were determined using a universal testing machine (Instron 5982, Instron, Norwood, MA, USA) in accordance with ASTM E8/E8M standards [37].

**Figure 3:**
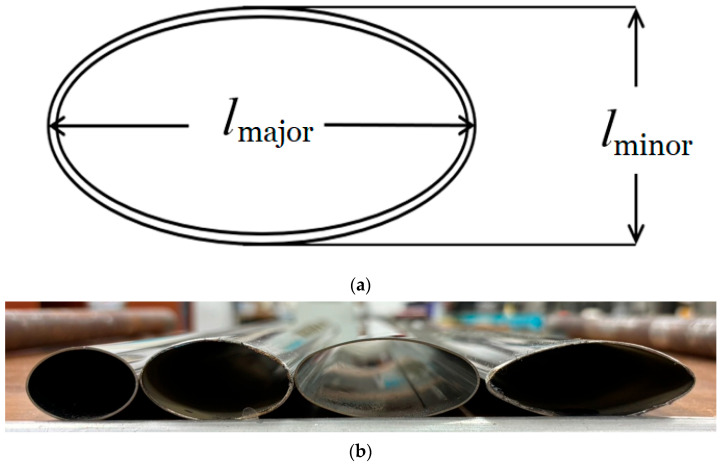
(**a**) Cross-sectional schematic of an elliptical tube showing major and minor axes, and (**b**) image of galvanized steel elliptical tubes with four different *ℓ*_maj_/*ℓ*_min_ ratios.

The elliptical tubes used in this study had a length of 500 mm and a uniform wall thickness of 0.7 mm. Figure 3a shows a schematic diagram defining the major and minor axes of the elliptical cross-section. Four major-to-minor axis ratios (*ℓ*_maj_/*ℓ*_min_) were investigated, as illustrated in Figure 3b: 1.5 (30 mm/20 mm), 2.0 (40 mm/20 mm), 2.5 (50 mm/20 mm), and 3.0 (60 mm/20 mm). These variations were selected to systematically examine the effects of cross-sectional geometry on bending response, minor-axis deformation, and instability behavior of elliptical tubes under cyclic loading.

### 2.4. Test Procedures

Elliptical tubes were subjected to curvature-controlled cyclic bending at a constant curvature rate of 0.05 m^−1^·s^−1^. The initial curvature range was set from −0.4 m^−1^ to +0.4 m^−1^ and was subsequently extended to −1.4 m^−1^ to +1.4 m^−1^ to investigate a wider range of bending deformations. The effect of mean curvature was characterized using the curvature ratio *r*, defined as the ratio of minimum-to-maximum curvature (*r* = minimum curvature/maximum curvature). Three values of *r* (−1, −0.5, and 0) were tested to assess the influence of mean curvature on the cyclic bending response.

The bending moment (*M*) was measured using load cells installed in the bending machine (Figure 1), while curvature (*κ*) and minor-axis variation (∆*ℓ*/*ℓ*_min_, where ∆*ℓ* denotes the change in the minor-axis length (*ℓ*_min_)) were recorded using the COMA system (Figure 2). The number of cycles to buckling (*N*_b_) was defined as the cycle at which the bending moment decreased by 20%, serving as a quantitative measure of structural degradation under repeated loading. This comprehensive dataset enabled a detailed analysis of the interaction between curvature, cross-sectional deformation, and cyclic instability in elliptical tubes.

## 3. Results and Discussion

This study investigates the mechanical behavior and buckling failure of galvanized steel elliptical tubes under cyclic loading. The tubes were fabricated with four outer major-to-minor axis length ratios (*ℓ*_maj_/*ℓ*_min_ = 1.5, 2.0, 2.5, and 3.0) and tested under three curvature ratios (*r* = −1, −0.5, and 0) to evaluate the combined effects of cross-sectional geometry and mean curvature on cyclic bending response. Based on the experimental results, relationships among bending moment (*M*)–curvature (*κ*), minor-axis variation (∆*ℓ*/*ℓ*_min_)–curvature (*κ*), and curvature range (Δ*κ*)–number of cycles to buckling (*N*_b_) are established. Finally, a theoretical equation is proposed to describe the Δ*κ*–*N*_b_ relationship.

### 3.1. Relationship Between Bending Moment (M) and Curvature (κ)

**Figure 4:**
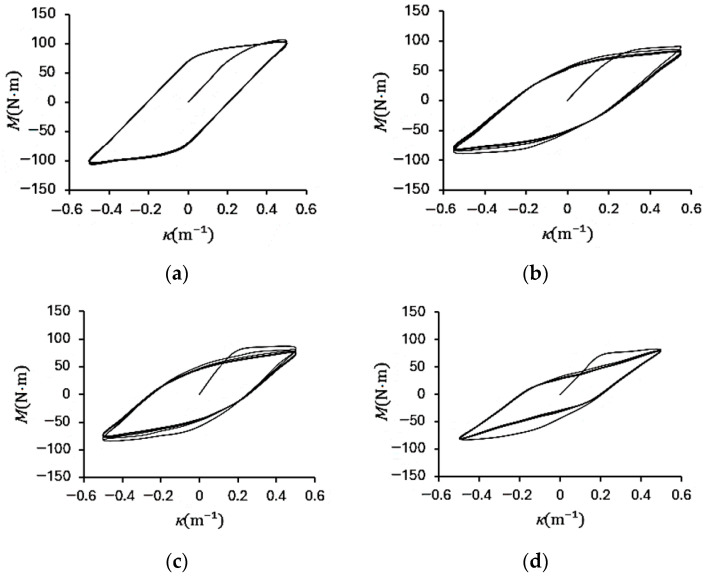
Experimental bending moment–curvature (*M*–*κ*) responses of galvanized steel elliptical tubes with *ℓ*_maj_/*ℓ*_min_ ratios of (**a**) 1.5, (**b**) 2.0, (**c**) 2.5, and (**d**) 3.0 subjected to cyclic curvature of ±0.5 m^−1^ (*r* = −1).

**Figure 5:**
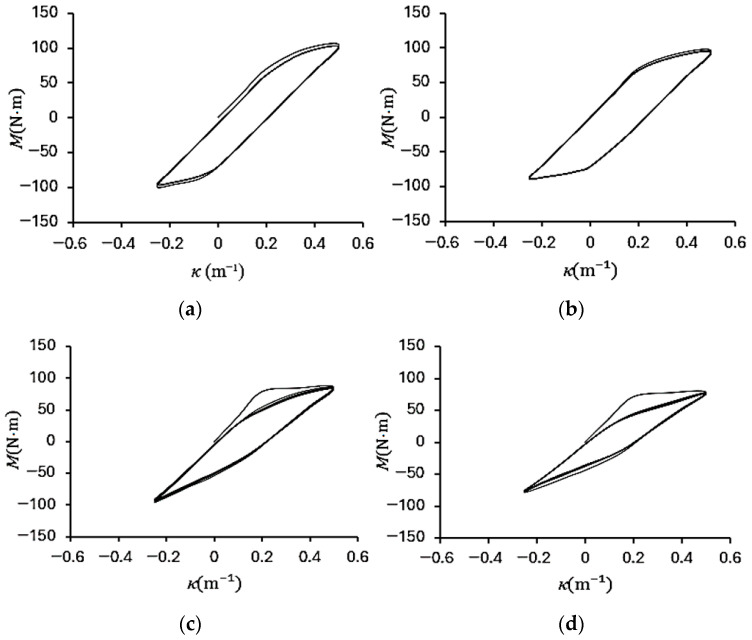
Experimental bending moment–curvature (*M*–*κ*) responses of galvanized steel elliptical tubes with *ℓ*_maj_/*ℓ*_min_ ratios of (**a**) 1.5, (**b**) 2.0, (**c**) 2.5, and (**d**) 3.0 subjected to cyclic curvature of +0.5~−0.25 m^−1^ (*r* = −0.5).

**Figure 6:**
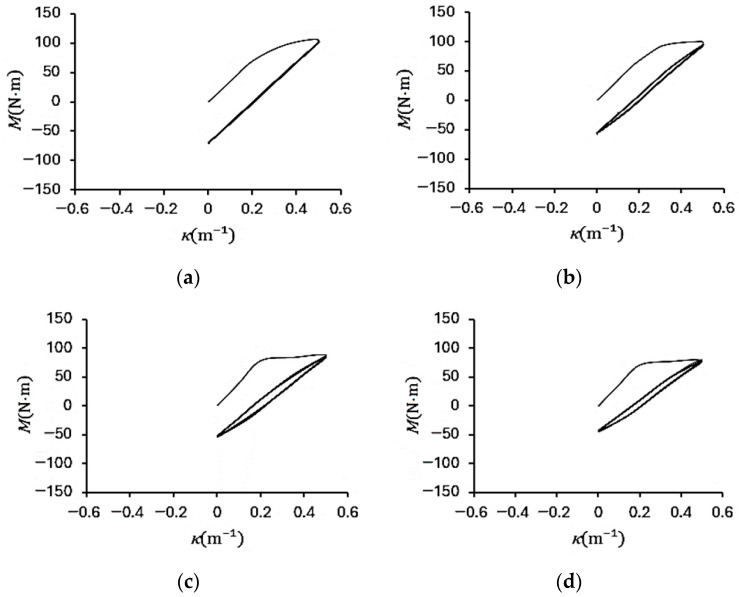
Experimental bending moment–curvature (*M*–*κ*) responses of galvanized steel elliptical tubes with *ℓ*_maj_/*ℓ*_min_ ratios of (**a**) 1.5, (**b**) 2.0, (**c**) 2.5, and (**d**) 3.0 subjected to cyclic curvature of +0.5~0 m^−1^ (*r* = 0).

Figure 4a–d show the experimental bending moment–curvature (*M*–*κ*) responses of galvanized steel elliptical tubes with *ℓ*_maj_/*ℓ*_min_ ratios of 1.5, 2.0, 2.5, and 3.0, subjected to cyclic curvature of ±0.5 m^−1^ (*r* = −1). Figure 5a–d present the corresponding *M*–*κ* responses for specimens tested under cyclic curvature ranging from +0.5 m^−1^ to −0.25 m^−1^ (*r* = −0.5), while Figure 6a–d show the results obtained under cyclic curvature ranging from 0.5 m^−1^ to 0 m^−1^ (*r* = 0).

For all curvature ratios, the *M*–*κ* loops gradually evolve into stable elastoplastic hysteresis loops as the number of cycles increases, indicating the establishment of a steady-state cyclic response. At a given curvature ratio, the maximum bending moment systematically decreases with increasing *ℓ*_maj_/*ℓ*_min_ ratio, reflecting the reduction in cross-sectional stiffness associated with more elongated elliptical geometries. Furthermore, for *r* = −0.5 and *r* = 0, the *M*–*κ* loops exhibit slight cyclic relaxation in the early stages of loading but ultimately converge to a consistent hysteretic behavior, demonstrating that the tubes achieve a stabilized elastoplastic response under repeated curvature-controlled bending.

### 3.2. Relationship Between Minor-Axis Variation (Δℓ/ℓ_min_) and Curvature (κ)

**Figure 7:**
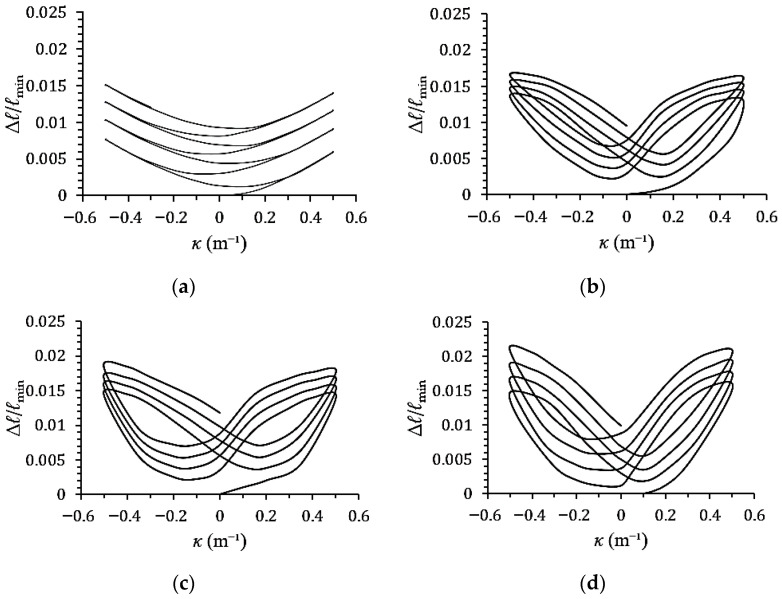
Experimental minor-axis variation–curvature (∆*ℓ*/*ℓ*_min_–*κ*) responses of galvanized steel elliptical tubes with *ℓ*_maj_/*ℓ*_min_ ratios of (**a**) 1.5, (**b**) 2.0, (**c**) 2.5, and (**d**) 3.0 subjected to cyclic curvature of ±0.5 m^−1^ (*r* = −1).

**Figure 8:**
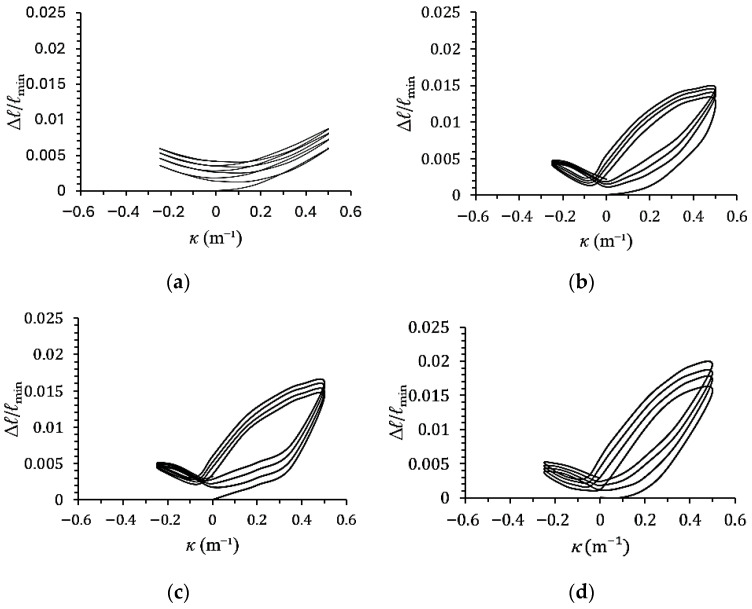
Experimental minor-axis variation–curvature (∆*ℓ*/*ℓ*_min_–*κ*) responses of galvanized steel elliptical tubes with *ℓ*_maj_/*ℓ*_min_ ratios of (**a**) 1.5, (**b**) 2.0, (**c**) 2.5, and (**d**) 3.0 subjected to cyclic curvature of +0.5~−0.25 m^−1^ (*r* = −0.5).

**Figure 9:**
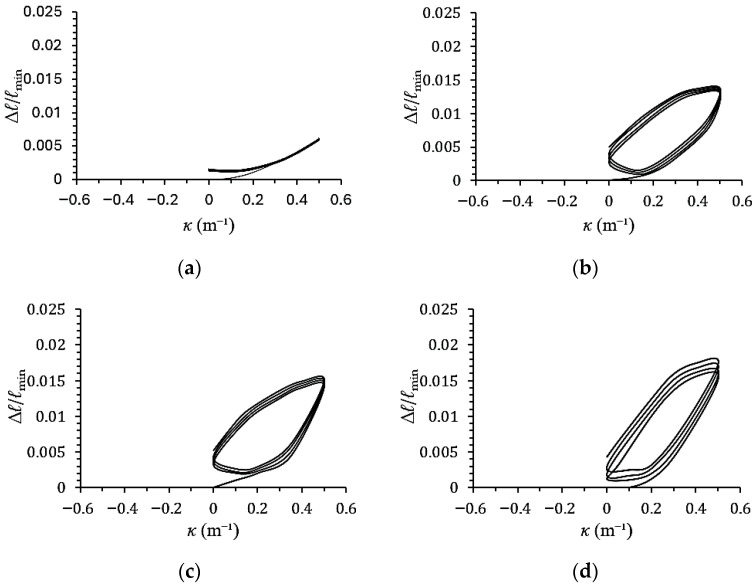
Experimental minor-axis variation–curvature (∆*ℓ*/*ℓ*_min_–*κ*) responses of galvanized steel elliptical tubes with *ℓ*_maj_/*ℓ*_min_ ratios of (**a**) 1.5, (**b**) 2.0, (**c**) 2.5, and (**d**) 3.0 subjected to cyclic curvature of +0.5~0 m^−1^ (*r* = 0).

Figure 7a–d show the experimental minor-axis variation–curvature (∆*ℓ*/*ℓ*_min_–*κ*) responses of galvanized steel elliptical tubes with *ℓ*_maj_/*ℓ*_min_ ratios of 1.5, 2.0, 2.5, and 3.0, subjected to cyclic curvature of ±0.5 m^−1^ (*r* = −1). Figure 8a–d present the corresponding ∆*ℓ*/*ℓ*_min_–*κ* responses for specimens tested under cyclic curvature ranging from +0.5 m^−1^ to −0.25 m^−1^ (*r* = −0.5), while Figure 9a–d show the results obtained under cyclic curvature ranging from 0.5 m^−1^ to 0 m^−1^ (*r* = 0).

Experimental results indicate that ∆*ℓ*/*ℓ*_min_ increases progressively with the number of loading cycles for all values of *r* and *ℓ*_maj_/*ℓ*_min_. When *r* = −1, the ∆*ℓ*/*ℓ*_min_–*κ* curves exhibit symmetric behavior, while at *r* = −0.5 and 0, the curves become asymmetric and shift toward the direction of the mean curvature. For *ℓ*_maj_/*ℓ*_min_ = 1.5, the ∆*ℓ*/*ℓ*_min_–*κ* relationship displays a serrated pattern due to the cross-section being closer to circular, thus resembling the behavior of circular tubes. In contrast, for *ℓ*_maj_/*ℓ*_min_ = 2.0, 2.5, and 3.0, the ∆*ℓ*/*ℓ*_min_–*κ* curves exhibit a butterfly-shaped pattern. Additionally, for a fixed curvature ratio *r*, a higher *ℓ*_maj_/*ℓ*_min_ ratio results in a greater increase in ∆*ℓ*/*ℓ*_min_.

### 3.3. Relationship Between the Curvature Range (Δκ) and Number of Cycles to Buckling (N_b_)

**Figure 10:**
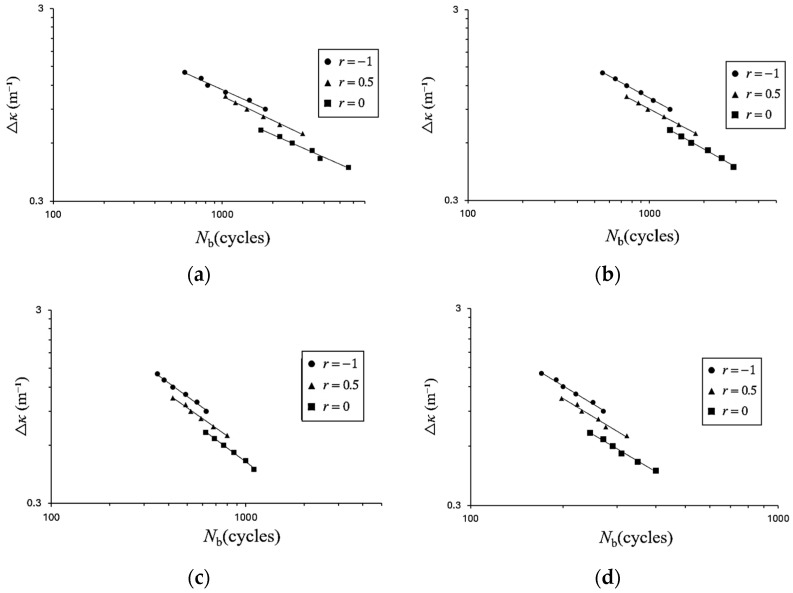
Experimental Δ*κ*–*N*_b_ responses of galvanized steel elliptical tubes with *ℓ*_maj_/*ℓ*_min_ ratios of (**a**) 1.5, (**b**) 2.0, (**c**) 2.5, and (**d**) 3.0 subjected to cyclic bending under various *r* on double-logarithmic coordinates.

Figure 10a–d present the Δ*κ*–*N*_b_ relationships of galvanized steel elliptical tubes subjected to cyclic bending, with *ℓ*_maj_/*ℓ*_min_ ratios of 1.5, 2.0, 2.5, and 3.0, respectively, plotted on double-logarithmic scales for different curvature ratios. In each figure, three fitted lines correspond to the three values of *r*, all obtained using the least-squares method. The results indicate that, for a given *ℓ*_maj_/*ℓ*_min_ ratio, *N*_b_ decreases with increasing Δ*κ*. For a fixed Δ*κ*, *N*_b_ increases as *r* approaches −1. Furthermore, at a given *r*, *N*_b_ decreases with increasing *ℓ*_maj_/*ℓ*_min_ ratio. It is noteworthy that, for each Δ*κ* level, two specimens were tested, and the variation in *N*_b_ between repeated tests was limited; the difference in *N*_b_ between the two specimens was within 10%, indicating that the experimental results exhibit acceptable repeatability.

In 1987, Kyriakides and Shaw [2] established a *κ*/*κ*_d_–*N*_b_ empirical relationship for smooth circular tubes under cyclic bending, which can be expressed as follows:

*κ*/*κ*_d_ = *A*(*N*_b_)^−*α*^ (1)

or equivalently,

log*κ*/*κ*_o_ = log*A* − *α*log*N*_b_. (2)

Here, *κ*_d_ denotes a characteristic curvature introduced to nondimensionalize, defined based on the tube geometry, specifically as the wall thickness divided by the square of the outer diameter. The parameters *A* and *α* are material-dependent constants. In this relationship, A represents the value of *κ*/*κ*_d_ at *N*_b_ = 1, while *α* corresponds to the slope of the *κ*/*κ*_d_–*N*_b_ curve when plotted on double-logarithmic coordinates.

In the present study, elliptical tubes are employed, for which a unique definition of *κ*_d_ is not available. Moreover, the maximum and minimum curvatures are asymmetric for curvature ratios *r* = −0.5 and 0. Consequently, the original formulation is not directly applicable. To account for these differences, Equations (1) and (2) are modified by replacing *κ*/*κ*_d_ with the curvature range Δ*κ*, yielding

Δ*κ* = *C*(*N*_b_)^−*α*^ (3)

or

logΔ*κ* = log*C* − *α*log*N*_b_, (4)

where C is a fitting parameter analogous to A.

**Figure 11:**
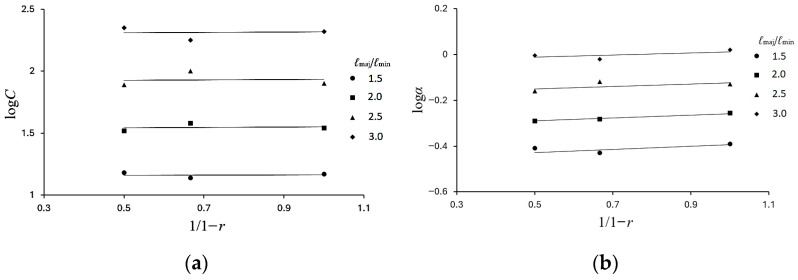
(**a**) Variation in log*C* with 1/(1 − *r*) for different *ℓ*_maj_/*ℓ*_min_ ratios; (**b**) variation in log*α* with 1/(1 − *r*) for different *ℓ*_maj_/*ℓ*_min_ ratios.

Based on the experimental results presented in Figure 10a–d, three sets of parameters *C* and *α* corresponding to the three curvature ratios *r* are determined using Equation (3). By examining the relationship between log*C* and 1/(1 − *r*), the relationship shown in Figure 11a is obtained. Similarly, the relationship between log*α* and 1/(1 − *r*) is illustrated in Figure 11b. In both figures, the data are well represented by straight lines fitted using the least-squares method. In view of the clear linear trends observed, the following relationships are proposed.

log*C* = *c*_1_(1/(1 − *r*)) + *c*_2_ (5)

and

log*α* = *a*_1_(1/(1 − *r*)) + *a*_2_, (6)

where *c*_1_, *c*_2_, *a*_1_, and *a*_2_ are material parameters.

**Figure 12:**
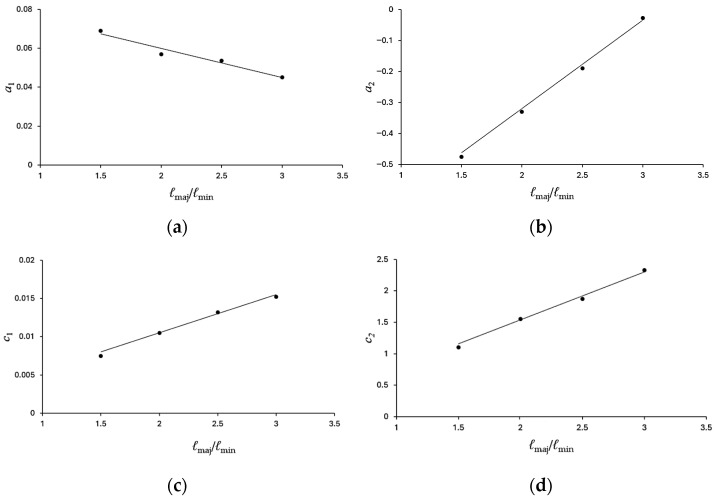
(**a**) Variation in *a*_1_ with *ℓ*_maj_/*ℓ*_min_; (**b**) variation in *a*_2_ with *ℓ*_maj_/*ℓ*_min_; (**c**) variation in *c*_1_ with *ℓ*_maj_/*ℓ*_min_; (**d**) variation in *c*_2_ with *ℓ*_maj_/*ℓ*_min_.

The material parameters *c*_1_, *c*_2_, *a*_1_, and *a*_2_ determined from Figure 11a,b are further extracted and correlated with the *ℓ*_maj_/*ℓ*_min_ ratio, as presented in Figure 12a–d. All four relationships exhibit clear linear trends. Accordingly, the following linear expressions are proposed to describe the dependence of *c*_1_, *c*_2_, *a*_1_, and *a*_2_ on *ℓ*_maj_/*ℓ*_min_.

*c*_1_ = *γ*_1_(*ℓ*_maj_/*ℓ*_min_) + *γ*_2_ (7)

*c*_2_ = *β*_1_(*ℓ*_maj_/*ℓ*_min_) + *β*_2_ (8)

*a*_1_ = *δ*_1_(*ℓ*_maj_/*ℓ*_min_) + *δ*_2_ (9)

and

*a*_2_ = *μ*_1_(*ℓ*_maj_/*ℓ*_min_) + *µ*_2_. (10)

**Figure 13:**
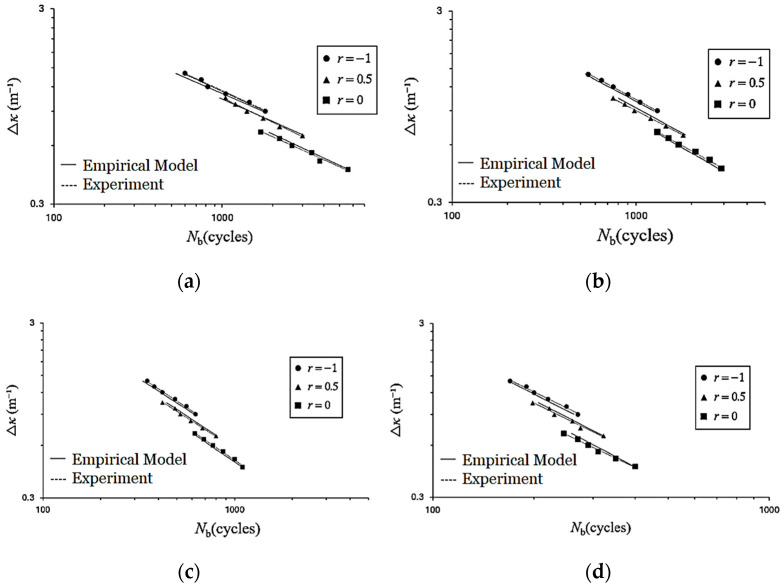
Experimental and descriptive Δ*κ*–*N*_b_ relationships of galvanized steel elliptical tubes with *ℓ*_maj_/*ℓ*_min_ = (**a**) 1.5, (**b**) 2.0, (**c**) 2.5, and (**d**) 3.0 under cyclic bending at different *r* on double-logarithmic coordinates.

**Figure 14:**
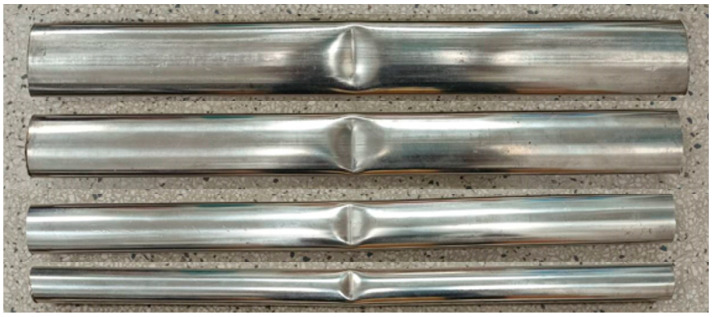
Photograph illustrating the buckling behavior observed in galvanized steel elliptical tubes.

Here, *γ*_1_, *γ*_2_, *β*_1_, *β*_2_, *δ*_1_, *δ*_2_, *μ*_1_ and *μ*_2_ are material parameters obtained from the linear analyses shown in Figure 12a–d. Their determined values are *γ*_1_ = 0.0051, *γ*_2_ = 0.0005, *β*_1_ = 0.7625, *β*_2_ = 0.0141, *δ*_1_ = −0.0153, *δ*_2_ = 0.091, *μ*_1_ = 0.2852, and *μ*_2_ = −0.8902. Using these parameters, Equations (4)–(10) are employed to predict the Δ*κ*–*N*_b_ responses of galvanized steel elliptical tubes with *ℓ*_maj_/*ℓ*_min_ ratios of 1.5, 2.0, 2.5, and 3.0 subjected to cyclic bending under various *r*. The predicted results are presented as solid lines in Figure 13a–d. A comparison between the predicted results and the experimental data demonstrates good agreement, indicating that the proposed empirical model effectively characterizes the observed cyclic bending behavior. Figure 14 presents a photograph illustrating buckling in galvanized steel elliptical tubes, highlighting specimens with four different *ℓ*_maj_/*ℓ*_min_ ratios at *r* = −0.5.

## 4. Conclusions

This study systematically investigated the combined effects of the major-to-minor axis length ratio (*ℓ*_maj_/*ℓ*_min_) and curvature ratio (*r*) on the cyclic bending response and buckling behavior of galvanized steel elliptical tubes. Particular attention was given to the evolution of minor-axis deformation, the relationship between curvature range (Δ*κ*) and the number of cycles to buckling (*N*_b_), and the development of a predictive empirical model. Based on the experimental and analytical results, the following conclusions can be drawn:

(1) The cyclic *M*–*κ* responses exhibited stable elastoplastic hysteresis loops after several loading cycles. For a given curvature ratio *r*, the peak bending moment decreased with increasing *ℓ*_maj_/*ℓ*_min_, demonstrating the significant influence of cross-sectional geometry on bending resistance and cyclic stability.
(2) The evolution of minor-axis variation strongly depended on both *ℓ*_maj_/*ℓ*_min_ and *r*. Symmetric ∆*ℓ*/*ℓ*_min_–*κ* behavior was observed at *r* = −1, whereas asymmetric responses developed at *r* = −0.5 and 0. Larger *ℓ*_maj_/*ℓ*_min_ ratios resulted in greater minor-axis variation, indicating enhanced ovalization and geometric instability effects.
(3) A clear power-law relationship was identified between Δ*κ* and *N*_b_. For fixed *ℓ*_maj_/*ℓ*_min_ and *r*, *N*_b_ decreased with increasing Δ*κ*. Moreover, increasing *ℓ*_maj_/*ℓ*_min_ or *r* led to a reduction in *N*_b_, confirming the combined geometric and loading effects on cyclic buckling resistance.
(4) A predictive empirical model was developed to characterize the Δ*κ*–*N*_b_ relationship. The proposed equations, derived from systematic regression analyses, show good agreement with experimental data, demonstrating their ability to effectively capture the cyclic degradation and instability behavior of galvanized steel elliptical tubes.

## Figures and Tables

**Table 1:** Chemical composition of the Q235 ordinary carbon structural steel (weight%).

Element	Fe	C	Si	Mn	P	S
**Proportion (%)**	98.85	0.22	0.35	0.48	0.05	0.05

**Table 2:** Chemical composition of the Z120 high-purity galvanized coating (weight%).

Element	Zn	Fe	Pb	Sn	Cd
**Proportion (%)**	99.76	0.12	0.01	0.002	0.002

**Table 3:** Mechanical properties of the galvanized steel.

Density	Elastic Modulus	Poisson’s Ratio	Yield Strength	Ultimate Strength
7833 $\mathrm{Kg}/m^{3}$	210 GPa	0.31	240 MPa	400 MPa

## Data Availability

The original contributions presented in this study are included in the article. Further inquiries can be directed to the corresponding author.
